# Estimating outflow facility parameters for the human eye using hypotensive pressure-time data

**DOI:** 10.1371/journal.pone.0238146

**Published:** 2020-08-25

**Authors:** David W. Smith, Chang-Joon Lee, Bruce S. Gardiner

**Affiliations:** 1 Faculty of Engineering and Mathematical Sciences, The University of Western Australia, Crawley, Australia; 2 College of Science, Health, Engineering and Education, Murdoch University, Murdoch, Western Australia, Australia; University of Nottingham, UNITED KINGDOM

## Abstract

We have previously developed a new theory for pressure dependent outflow from the human eye, and tested the model using experimental data at intraocular pressures above normal eye pressures. In this paper, we use our model to analyze a hypotensive pressure-time dataset obtained following application of a Honan balloon. Here we show that the hypotensive pressure-time data can be successfully analyzed using our proposed pressure dependent outflow model. When the most uncertain initial data point is removed from the dataset, then parameter estimates are close to our previous parameter estimates, but clearly parameter estimates are very sensitive to assumptions. We further show that (i) for a measured intraocular pressure-time curve, the estimated model parameter for whole eye surface hydraulic conductivity is primarily a function of the ocular rigidity, and (ii) the estimated model parameter that controls the rate of decrease of outflow with increasing pressure is primarily a function of the convexity of the monotonic pressure-time curve. Reducing parameter uncertainty could be accomplished using new technologies to obtain higher quality datasets, and by gathering additional data to better define model parameter ranges for the normal eye. With additional research, we expect the pressure dependent outflow analysis described herein may find applications in the differential diagnosis, prognosis and monitoring of the glaucomatous eye.

## Introduction

Glaucoma is the most significant cause of irreversible blindness world-wide, with some 70 million people affected [1]. While glaucoma is a group of diseases, there is a crucially important association between the initiation and progression of glaucoma and raised intraocular pressure (IOP). It is hypothesized that raised IOP can directly lead to optic nerve neuropathy. The only proven treatment of glaucoma is reduction of IOP [1, 2].

Given the importance of IOP from diagnostic, monitoring and prognostic viewpoints, trying to measure and understand the causes of elevated IOP has attracted considerable attention. It is known that IOP is determined by the interplay between net fluid production and pressure dependent outflow from the eye. Impairment of outflow facility of the eye is believed to be the primary determinant of raised IOP [3]. Consequently there is considerable clinical interest in measuring the outflow facility, both to identify a primary risk for glaucoma and to monitor the response to interventions, such as drug treatments.

Outflow occurs through the trabecular meshwork (the so-called ‘conventional pathway’) and through the uveoscleral route (the so-called ‘unconventional pathway’) [4]. But it is also well-documented that pressure dependent and pressure independent outflow also occurs across the retinal pigmented epithelium and into the choriocapillaris [5–16]. This latter pathway is often neglected in discussions of eye fluid turnover [2, 4, 17], probably because it is difficult to measure clinically [18].

In a previous paper we developed a new theoretical model for the analysis of pressure dependent outflow analysis for the *in vivo* eye [19], which includes outflow across the retinal pigment epithelium [16]. To unambiguously develop the pressure dependent outflow theory requires greater precision in the way outflow facility (*C*) is defined [20]. Indeed, when outflow facility is pressure dependent, there are any number of outflow facilities that can be measured for an eye. For this reason, we introduced a new notation that removes ambiguity about precisely which outflow facility is being considered or measured. That is, we define two distinct quantities:

- a local (or point) estimate of outflow facility, which is the mathematical derivative of the total pressure dependent outflow with respect to IOP (denoted $C_{}^{p_{1}}$ at IOP *p*_1_). For example *C*^15^ (where *C*^15^ means the derivative of the total pressure dependent outflow with respect to IOP evaluated at an IOP of 15 mm Hg). This is the tangent to the outflow versus pressure curve at a specific pressure;
- an average of local outflow facility over a pressure range (denoted ${\bar{C}}_{p_{1}}^{p_{2}}$ over a pressure range *p*_1_ to *p*_2_). For example ${\bar{C}}_{15}^{20}$ (where the overbar denotes average, and ${\bar{C}}_{15}^{20}$ means the average of the local outflow facilities over the IOPs ranging from 15 mm Hg to 20 mm Hg). This is the slope of the secant line joining two points for the two pressures on an outflow versus pressure curve.

The clinically measured outflow facility corresponds to the second definition, and represents an average outflow facility across a pressure range (typically from the initial eye pressure up to an elevated eye pressure). For completeness, we briefly describe the governing equations for the pressure-dependent model using this new nomenclature. Employing published experimental data, we then solve the model equations to estimate two key parameters governing outflow in the model, namely: (i) the hydraulic outflow conductance for the whole eye, $C_{T}^{SL}$ (μl/min/mm Hg), describing membrane outflow properties; and (ii) an exponential decay constant *α* (mm Hg)^-1^ for the whole eye, which describes the rate of decrease of local outflow facility with increasing IOP (mm Hg).

In contrast to our previous paper [19], in this paper we solve the pressure dependent outflow equations to make estimates of parameters $C_{T}^{SL}$ and *α* at IOPs below the normotensive pressure. This approach provides another independent dataset to test the robustness of our previous parameter estimates of $C_{T}^{SL}$ and *α*. To do this we employ published experimental data collected for the purpose of investigating the time course of ocular hypotony following application of a ‘Honan intraocular pressure reducer’ [21]—sometimes referred to as a ‘Honan balloon’ [22]. Based on this set of experimental measurements, we again estimate a total pressure dependent outflow for aged but otherwise healthy *in vivo* human eyes. Our analysis reveals some important findings. For example: (i) that for a given IOP-time curve, the parameter $C_{T}^{SL}$ is primarily a function of the ocular rigidity, and (ii) the parameter *α* is primarily a function of the shape of the monotonic pressure-time curve.

We begin our analysis by first briefly outlining our pressure dependent outflow theory required for the analysis, and then describe our strategy to combine computational modeling and statistical analysis of the Ernest et al.’s experimental data [21], to uncover the most likely mean pressure time curve following release of the Honan balloon. We use minimization of variance to estimate the most likely model parameters, and then explore parameter estimation upon removal of the initial data point, and for given additional constraints on outflow parameters. While our previous analysis [19] was largely based on pressure-time data above normotensive pressures, the analysis reported here is based on pressure-time data below normotensive pressures. There is reason to suppose that analysis of the data for these two pressure ranges should result in reasonably similar outflow predictions for the human eye. In proceeding along this investigation path, considerable effort will be given to comparing multiple data fitting strategies, motivated either by mathematical or experimental limitations.

## Method

### Theory for the analysis of pressure-volume and pressure-time measurements

The average pressure dependent outflow facility over a pressure range *p*_1_ to *p*_2_, denoted ${\bar{C}}_{p_{1}}^{p_{2}}$, is the average of the local outflow facilities between pressures *p*_1_ and *p*_2_. This quantity turns out to be a well-known ratio of finite differences [23, 24], viz,

$${\bar{C}}_{p_{1}}^{p_{2}}=\frac{1}{p_{2}-p_{1}}\int_{p_{1}}^{p_{2}}C^{p}dp=\frac{1}{p_{2}-p_{1}}\int_{p_{1}}^{p_{2}}\frac{d{\dot{V}}_{out}}{dp}dp=\frac{{\dot{V}}_{out}(p_{2})-{\dot{V}}_{out}(p_{1})}{p_{2}-p_{1}}$$ (1)

where ${\dot{V}}_{out}(p_{2})$ is outflow rate at *p*_2_, and similarly for the outflow rate at *p*_1_. If the finite difference is small, then Eq (1) can be employed to approximate $C_{}^{p_{1}}$, for as *P*_2_ → *P*_1_ so ${\bar{C}}_{p_{1}}^{p_{2}}\to C_{}^{p_{1}}$. An incremental volume flow rate into the eye may be held constant until the IOP stabilizes at a new level above the resting IOP, and the average outflow facility over this pressure range may then be calculated directly using Eq (1). Very occasionally this technique has been employed using *in vivo* human eyes [25].

More often for human eyes, outflow facility is estimated from recordings of a pressure-volume curve and a pressure-time curve for an individual eye. Direct cannulation of the eye is the most accurate method. In this approach, one first volumetrically loads the eye with fluid and records IOP, so measuring the pressure-volume curve for the eye. Then volumetric loading of the eye is ceased and the change in pressure over time is manometrically measured (obtaining the ‘pressure-time’ curve) [26, 27]. When pressure-time and pressure-volume data is available, the outflow facility between the normotensive intraocular pressure (*p*_nt_) and some other pressure *p* (${\bar{C}}_{nt}^{p}$) may also be calculated using the following equation from Ref [19],

$${\bar{C}}_{nt}^{p}=\frac{{-\frac{d\Delta V_{e}}{dt}|}_{p}}{p-p_{nt}}=\frac{-(\frac{c_{0}}{p}+c_{1}){\frac{dp}{dt}|}_{p}}{p-p_{nt}}$$ (2)

where *p*_*nt*_ is the normotensive pressure, ${\frac{d\Delta V_{e}}{dt}|}_{p}$ is the rate of change of the increment of volume with respect to time at some pressure *p*. Note that to derive Eq (2), we have used the chain rule of differentiation ($\frac{d\Delta V_{e}}{dp}\frac{dp}{dt}=\frac{1}{Kp}\frac{dp}{dt}$) and introduced the pressure-volume constitutive relationship for the eye [19],

$$\frac{1}{K}=c_{0}+c_{1}p$$ (3)

where *K* is known as the ocular rigidity, and *c*_0_ and *c*_1_ are two experimental estimated constants estimated by fitting the pressure-volume curve. It is apparent that an accurate estimation of the ocular rigidity is very important for the accurate estimation of average outflow facility between two IOPs from a pressure-time curve, as the ocular rigidity determines the eye fluid volume change associated with a pressure change. Rearranging Eq (2) leads to,

$${\frac{dp}{dt}|}_{p}=-\frac{{\bar{C}}_{nt}^{p}p(p-p_{nt})}{c_{0}+c_{1}p}$$ (4)

which governs the pressure-time response behavior for a human eye system with no external fluid injection.

### Experimental data to be analyzed

Ernest et al. [21] investigated the ‘softening of the eye’ arising from a reduction in IOP, which can be achieved following application of the ‘Honan intraocular pressure reducer’ (also known as a Honan balloon). A softened eye is deemed desirable by surgeons prior to eye surgery, for it is believed that sudden pressure release and associated volume change of the eye that occur upon cutting into a ‘hard’ eyeball, may potentially displace vitreous humor.

In Ernest et al. [21], 10 subjects waiting for cataract surgery, were examined and their eyes deemed otherwise healthy [21]. All subjects were 50+ years of age. IOP was measured using a Goldmann applanation tonometer, and Ernest et al. demonstrated in one individual that they could achieve a good level of reproducibility over seven tonometer measurements, reporting a standard error of ± 0.2 mm Hg for the seven measurements. The average initial eye pressure for the 10 subjects prior to inflating the Honan balloon was reported to be 17.0 ± 1.4 mmHg [21].

**Fig 1:**
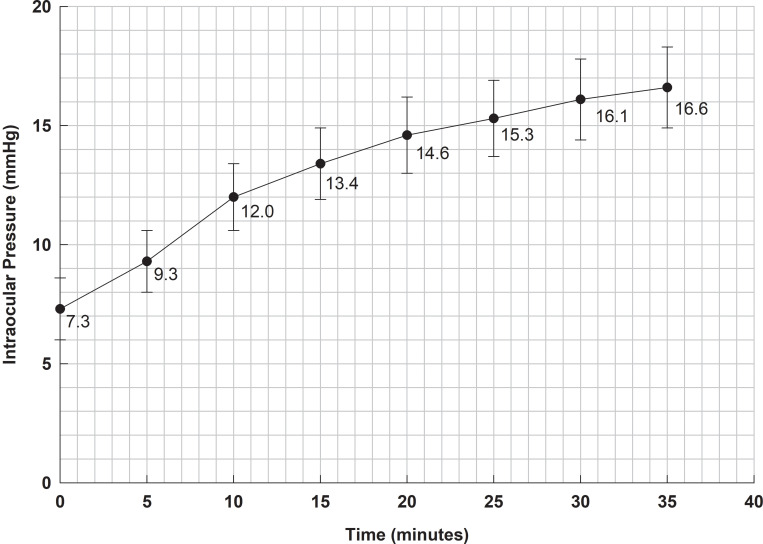
Data for the IOP recovery, measured at 5 minute intervals following removal of the Honan balloon [21]. Data points shown equal the mean value of measurements made on 7 to 10 subjects at each 5 minute interval (mean values shown have been estimated from the graph shown in fig *4*of Ernest et al.). Magnitude of error bars equal to ± 1 standard deviation for individual measurements (standard deviations shown have been estimated from graph in fig *4*of Ernest et al.). The mean IOP prior to application of Honan balloon equals 17 ± 1.4 mm Hg.

For the duration of the experiment, subjects remained quietly seated in a ‘slit-lamp chair’. The eye was dilated with one drop of 10% phenylphrine hydrochloride applied once every 5 minutes for 15 minutes. The Honan balloon was placed over closed eyelids and inflated to a gauge reading of 30, which then remained in place for 30 minutes. The initial reading, designated time zero, was made within 10 seconds of removing the Honan balloon, and repeated every 5 minutes thereafter to 35 minutes. The reported mean IOP for the subjects, and standard deviations of the individual measurements, is reproduced in Fig 1 below [21].

**Fig 2:**
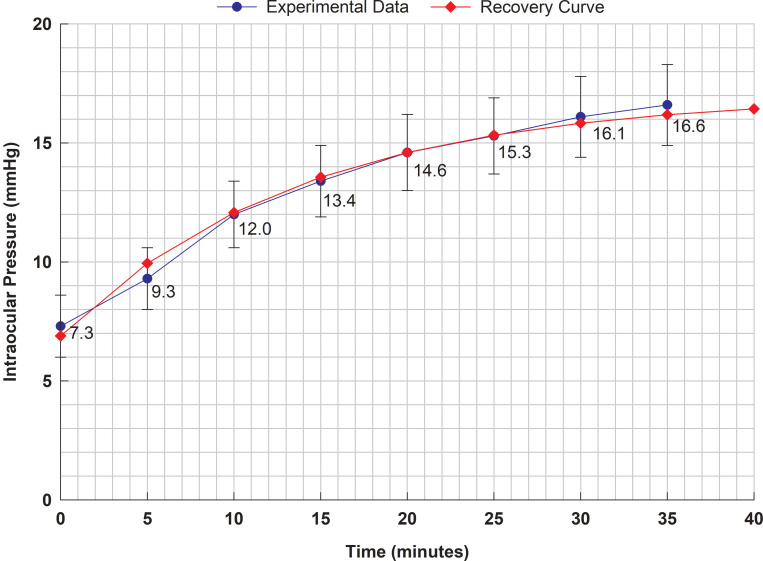
Exponential ‘recovery curve’ (red) fitted to experimental pressure data (blue), following removal of a Honan balloon [21]. Half-life for recovery t_1/2_ reported by Ernest et al. to be 9.6 minutes, while we calculate 9.81 minutes (this regression curve and half-life is calculated by directly minimizing the variance of experimentally measured and regression curve predicted means). We estimate the equation describing the recovery curve to be 17.0–10.1×EXP(-0.0707×t) mm Hg. Error bars shown in the figure are 95% confidence intervals for the mean data values (i.e. about 2.2 × SEM, which is calculated to be about ± 0.8 mm Hg initially, rising to ± 1.1 mm Hg at the final data point). The fitted exponential curve is seen to pass within the 95% confidence interval of each mean data point. We note that the exponential curve shown above looks very similar to that shown in Ernest et al. [21].

Based on an exponential curve fitted to the data presented in Fig 1, the half-time for pressure recovery was reported by Ernest et al. to be 9.6 minutes [21]. Our exponential curve fitted to the data in Fig 1 is shown in Fig 2, and the half-life for pressure recovery was found to be 9.8 minutes.

### Method of data analysis

To analyze Ernest et al.’s data shown in Fig 1, we have employed a combination of statistical regression modeling (based on minimizing variance between cubic polynomial and exponential regression curves and the experimental data) and computational modeling (based on minimizing variance between the theoretical outflow model (i.e. Eq (4)) and the experimental data). We know from the theoretical model that the outflow parameters depend upon accurate representations of the time derivative of the pressure-time and the pressure-volume data. However pressure-volume data was not measured by Ernest et al., so for our estimation of outflow parameters using Eq (4) it is necessary for us to estimate the ocular rigidity. To do this we employ estimates of ocular rigidity measured in similar groups of subjects (i.e. subjects of similar age also awaiting cataract surgery, and with an otherwise healthy eyes), as reported in the high quality clinical studies by Dastiridou et al. and Karyotakis et al. [26, 27].

The accurate representation of the time derivative of the mean pressure-time curve is challenging due to the uncertainty in the measured mean values. This uncertainty arises from both subject variability and measurement error. One approach to reducing uncertainty is to select a suitable regression curve to interpolate the experimental data by fitting a smooth curve through the data points.

Regression analysis involves a trade-off between decreasing the number of parameters in the regression model and having a comparatively poor model fit to the data points versus increasing the number of parameters in the regression model and improving the model fit to the data points. If the regression model has too few parameters, the regression model may be over-constrained (i.e. the regression curve is not ‘flexible’ enough), which can result in poor approximation to the time derivative of the pressure. For example, if the regression model is a traditional two parameter linear regression, the time derivative of the pressure would be a constant value. But the obvious curvature of the pressure-time data shown in Figs 1 and 2 means that the time derivative of the pressure is not a constant. Therefore a two-parameter linear regression model is not suitable for our purposes.

Ernest et al. employed a two-parameter exponential regression model, and this yields quite a good fit to the data (e.g. see Fig 2), so this might be suitable regression model to use here. However this exponential regression model also only involves two parameters, and the time derivative of the pressure is not necessarily an exponential curve. To see this, we note that if the theoretical model is correct, and assuming *c*_1_ is zero, then Eq (4) suggests that the time derivative of the pressure-time curve is actually a product of an exponential function of pressure (${\bar{C}}_{nt}^{p}$) and a quadratic polynomial in pressure (*p*(*p*−*p*_*nt*_)) [19].

We initially focus on a polynomial regression model to represent the pressure-time curve. If the time derivative is represented by a quadratic polynomial, the pressure-time curve is itself a cubic polynomial. This led us to considering cubic and higher order polynomial regressions. However given there is only eight data points collected at five minute intervals over 35 minutes, there is a significant danger of overfitting the data as the order of the polynomial increases. Taking this into account, we chose to initially use a cubic polynomial regression to model the pressure-time data, which means we use a quadratic polynomial to represent the time derivative of the pressure. We can see from Fig 2, an exponential can give a reasonably good fit to the data, so we will also check how an exponential curve performs in regression.

Our modeling strategy is to first directly fit the computational model (based on solving the ordinary differential equation shown in Eq (4)) to the raw experimental data, choosing the optimal fit based on minimizing the variance between computational model prediction and the experimental data. If the theoretical model is an accurate representation of the eye’s outflow system, then this may fit the experimental data better than either a cubic polynomial or an exponential regression. Then postulating the likelihood of a temporary systematic error associated with the first data point, we then show the computational model variance (i.e. the sum of squares of residuals) can be improved by removing the experimental data point at time zero from the optimization process, and that the predicted new intercept at time zero supports the existence of a temporary systematic error.

Second we fit the cubic polynomial regression model to the raw data, and then fit the computational model (based on Eq (4)) to this regression. We also do this ‘double fitting’ procedure for an exponential regression, and then evaluate if this double fitting approach is useful. This double fitting procedure has the potential advantage of smoothing the raw data before the computational model fitting, but has the potential disadvantage of introducing two lots of modeling error.

Both the statistical model regressions and the computational model regressions are computed in Excel, and models are fitted to the data using the GRG non-linear optimization routine (precision in the optimization solver was set to 1.0×10^−6^). Optimization is based on minimizing the ‘standard error of the regression’ (denoted $S=\sqrt{\sum e^{2}/df}$, where *e* is the error and *df* are the degrees of freedom), and this is applicable to both linear and non-linear regression models.

We note that ± the two-sided ‘t factor’ for the 95% confidence interval (*t*_*α*=0.95,*df*_) multiplied by the standard error of the regression is approximately the 95% confidence interval for the mean predictions. The cubic polynomial regression model has four fitting parameters including the intercept (i.e. 8 data points and 4 degrees of freedom, so the ‘*t* factor’ (*t*_*α*=0.95,4_) for the 95% confidence interval of a *t* distribution is about 2.78). The exponential regression model has two fitting parameters including the intercept (i.e. 8 data points and so 6 degrees of freedom, so the ‘*t* factor’ (*t*_*α*=0.95,6_) for the 95% confidence interval of a *t* distribution is about 2.45). The computational model based on Eq (4) has three fitting parameters (the IOP at time zero (i.e. the intercept), the hydraulic conductance for outflow $C_{T}^{SL}$ and the exponential decay constant *α*), and three additional fixed parameters, namely the initial IOP and the ocular rigidity parameters *c*_0_ and *c*_1_ (i.e. 8 data points and so 5 degrees of freedom, so the ‘*t* factor’ (*t*_*α*=0.95,5_) for the 95% confidence interval of a *t* distribution is about 2.57).

For the optimization of the standard error of the regression, the computational model fitting parameters can take any value that is greater than zero. The initial IOP is held at the experimentally reported value of 17 mm Hg for all analyses. Ernest et al. did not measure ocular rigidity, so we consider two mean ocular rigidities reported for two groups of subjects of similar age and also awaiting cataract surgery, but otherwise healthy eyes): (i) Dastiridou et al. [26] (*c*_0_ equals 45.5 and *c*_1_ equals zero) and (ii) Karyotakis et al. [27] (*c*_0_ equals 35.3 and *c*_1_ equals zero).

All simulations were carried out using a Dell PC with an Intel Core i7 3.40-GHz CPU running Windows 7 Professional. The solution time for optimization of the computational model and regression model in Excel was typically less than thirty seconds, with the analysis time being primarily dependent on chosen initial values.

## Results

Abbreviations are employed extensively throughout the Results section, so all the abbreviations employed are collected together in Table 1 for convenience.

**Table 1:** Abbreviations: Abbreviations as shown in the table assume the ocular rigidity is *C*_0_ equals 45.5. Abbreviations for ocular rigidity *C*_0_ equals 35.3 have a ‘b’ added to those abbreviation (e.g. CMOb).

Data	Abbreviation
Original data set	ED0
Original data set with first data point removed	ED1
The ED1 data set with first data point replaced by the CM1 model prediction	ED1*
Model fit to the data sets	
Computational model fit to ED0	CM0
Computational model fit to ED1	CM1
Polynomial regression curve fit to ED0	PM0
Exponential regression curve fit to ED0	EM0
Polynomial regression curve fit to ED1	PM1
Exponential regression curve fit to ED1	EM1
Computational model fit to PMO predictions	PCM0
Computational model fit to PM1 predictions	PCM1
Computational model fit to EM0 predictions	ECM0
Computational model fit to EM1 predictions	ECM1

For the analysis, w first we took a straightforward approach and simply fitted our pressure dependent outflow model to the experimental data. In the following Tables, Ernest et al.’s data set (shown in Fig 1) is labelled ED0, and the computational model fit to the data is labelled CM0 (and CM0b). The predicted outflow parameter *α* for the solution CMO is greater than zero, which means the solution is pressure-dependent. However *α* has comparatively small magnitude (*α* equals 0.027) relative to later analyses (see Table 2).

**Table 2:** Outflows and parameter solutions (to Eq 4) predicted by the computational model (denoted CM and PCM), for two ocular rigidities. CMO is computational model fitted to data ED0. PCM0 is the computational model fitted to the cubic polynomial regression on ED0. CM1 is computational model fitted to ED1. PCM1 is computational model fitted to cubic polynomial regression on ED1. ECM1 is computational model fitted to exponential regression on ED1.

	CM0	CM0b	PCM0	PCM0b	CM1	CM1b	PCM1	PCM1b	ECM1	ECM1b
*c*_0_	45.5	35.3	45.5	35.3	45.5	35.3	45.5	35.3	45.5	35.3
$C_{T}^{SL}$	0.42	0.33	0.47	0.37	1.51	1.17	2.15	1.67	3.27	2.53
*α* mmHg^-1^	0.027	0.027	0.035	0.035	0.11	0.11	0.14	0.14	0.16	0.16
Outflow μl/min	4.5	3.5	4.7	3.7	7.6	5.9	9.0	7.0	11.0	8.5

Next we fit the experimental data set ED0 to a cubic polynomial regression curve, and this prediction is labelled PM0 (see Table 3). We then fit the computational model to the regression curve PM0, obtaining a prediction labelled PCM0 (and PCM0b) (see Table 2). This ‘double fitting’ procedure is explored to assess its usefulness. After fitting the computational model to the cubic polynomial regression curve, we see that the predicted outflow parameters for the computational model solution PCM0 (and PCM0b) are again pressure-dependent, but now the parameter values have increased somewhat (i.e. $C_{T}^{SL}$ is increased by 12% to 0.47 μl/min/ mm Hg, *α* by 30% to 0.035 mm Hg^-1^ and the predicted outflow is increased by 4% to 4.7 μl/min; see Table 2).

**Table 3:** Time variation of mean IOP predictions predicted by cubic polynomial regression model fitted to various data sets (denoted PM), and exponential regression model (denoted EM).

Time	n	ED0	PM0	EM0	ED1	PM1	EM1
		Expt data	prediction	prediction	Expt data	prediction	prediction
0	10	7.3	7.15	6.91	removed	5.89	5.52
5	10	9.3	9.70	9.92	9.3	9.36	9.31
10	10	12	11.75	12.02	12	11.83	11.85
15	10	13.4	13.33	13.50	13.4	13.51	13.55
20	10	14.6	14.55	14.55	14.6	14.61	14.69
25	8	15.3	15.45	15.28	15.3	15.36	15.46
30	8	16.1	16.10	15.79	16	15.97	15.97
35	7	16.6	16.57	16.15	16.6	16.65	16.31

The data sets are: EDO, the original data; ED1, the original data with the initial time point removed. PM0 is cubic polynomial regression model fitted to data set ED0, PM1 is the cubic polynomial fitted to data set ED1, and EM1 is an exponential regression model fitted to data set ED1. The number of subjects contributing to the calculated mean at each time point (n) is shown in the second column.

We next reasoned that the first measured data point is likely to be least accurate, because that measurement was taken within 10 seconds of removing the Honan balloon after having been in place for 30 minutes. We first observe that the measurement is not taken at time zero, and even a short delay may potentially lead to a significant error when the systems state is adjusting very quickly. Immediately following removal of the Honan balloon it seems likely the IOP would be increasing, which suggests the actual initial IOP may be lower than that measured.

Further we expect that, following removal of the Honan balloon, eye physiology would be temporarily abnormal. For example, there may be a short-term reactive hyperemia within the orbit (e.g. associated with the choroidal vasculature increasing volume upon pressure release), and/or there may be short-term adjustments in interstitial fluid volumes within the orbit (e.g. fluid being squeezed into eye tissues when the anterior, posterior and vitreous chambers at elevated IOP may reverse flow direction upon pressure release of the Honan balloon), and/or perhaps there is temporary abnormal autonomic activity, all which may directly or indirectly impact measured IOP. In addition, measurement using the Goldmann applanation tonometer within a 10 second time limit would no doubt have been challenging, and probably contributed to comparatively more measurement error, at least for the initial time point (though we note the standard deviation of for the initial data point is not increased relative to other data points with 10 measurements, suggesting measurement error remains significantly less than subject to subject variability). In conclusion, these biologically plausible reasons raise suspicion that the first experimental data point may be significantly less accurate than measurements at later times. Therefore, we then proceed to model the original data set with this initial measurement excluded from the model fitting process (this data set is referred to as the ‘reduced data set’, and labelled ED1 (see Table 3).

Following the same procedure as for the complete data set, we first fit the computational model to the reduced data set labelled ED1 (the computational model prediction is labelled CM1 in Table 2). We immediately observe that the optimal parameters and outflow have increased significantly again (i.e. $C_{T}^{SL}$ is increased by a further 320% to 1.51 μl/min/ mm Hg, *α* by a further 310% to 0.11 mm Hg^-1^ and the predicted outflow by 160% to 7.6 μl/min; see Table 2).

At this stage we can gain insight into the appropriateness of removing the first data point from the computational model fitting procedure. To do this we calculate the variances for each data point in the complete data set (EDO) relative to computational model predictions CM1 (see variances under column labelled Var CM1-ED0; see Table 4).

**Table 4:** Variance (denoted Var; square of the residual) between optimized solution and data set.

Time (min)	Var CM0-ED0	Var PM0-ED0	Var PCM0-PM0	Var CM1-ED1	Var CM1-ED0	Var PM1-ED1	Var PM1-ED0	Var EM1-ED1	Var PCM1-PM1	Var ECM1-EM1
0	0.0067	0.022	0.0041	NA	0.90	NA	2.16	NA	0.0012	0.00020
5	0.073	0.16	0.013	0.0045	0.0045	0.0028	0.0043	0.0034	0.0062	0.0021
10	0.088	0.065	0.00058	0.047	0.047	0.023	0.00025	0.030	0.00015	0.00069
15	0.00011	0.0038	0.0056	0.013	0.013	0.014	0.00091	0.0097	0.0018	0.0011
20	0.0030	0.0025	0.0087	0.0089	0.0089	1.5E-10	0.0080	0.00089	0.0089	0.00014
25	0.042	0.022	0.0015	0.034	0.034	0.00057	0.016	0.0092	0.013	8.8E-05
30	0.0013	0.00	0.0032	0.0091	0.0091	0.0058	0.00099	0.0074	0.00022	0.00054
35	0.033	0.0011	0.028	0.064	0.064	0.0011	0.11	0.11	0.11	0.00087
Sum Var	0.25	0.28	0.064	0.18	1.08	0.048	2.30	0.17	0.14	0.0056
*S*	0.22	0.27	0.11	0.19	0.52	0.11	0.67	0.17	0.17	0.034

The data sets are: EDO, the original data; ED1, the reduced data set is the original data with the initial time point removed. *S* denotes standard error of the regression.

This analysis reveals that 83% (0.9/1.08) of the total variance (total variance being the sum of the variances for all 8 data points) is now explained by the first data point, while the other seven data points explain the remaining 17% (i.e. average 2.5% per data point). This degree of focusing of variance on a single data point is not nearly so marked if repeated after removal of other single data points (e.g. the variance explained if the second data point is removed is 60%). This suggests that the initial data measurement is consistent with an imputed systematic error of around 1.0 mm Hg, based upon fitting the computation model to the ED1 data set (CM1) (i.e. 7.3 mm Hg (EDO) minus 6.4 mm Hg (CM1 prediction); see Table 5).

**Table 5:** Estimated mean intercepts when models are fitted to the reduced data set ED1.

Expts and Model Type	Mean estimate of intercept (mm Hg)	SEM or *S* (mm Hg)	*t*_*α*=0.95,4_	95% confidence interval for prediction ≈ mean ± *t*_*α*=0.95_*(SEM or *S*)
Experimental	7.3	0.39	2.2	6.4–8.2
CM1	6.4	0.19	2.6	5.9–6.9
PM1	5.9	0.11	2.8	5.6–6.2
EM1	5.5	0.17	2.5	5.1–5.9

CM1 is computational model fitted to ED1; PM1 is the cubic polynomial fitted to data set ED1, and; EM1 is an exponential regression model fitted to data set ED1.

Fitting the regression curves also imputes a significant systematic error associated with the first data point, demonstrating that this observation is not specific to the computational model. For example, when data ED1 is regressed using a cubic polynomial, 94% of the variance to ED0 is focused on the first data point, and the imputed systematic error is somewhat larger at around 1.4 mm Hg (i.e. 7.3 mm Hg (EDO) minus 5.9 mm Hg (PM1)). If data set ED1 is regressed using an exponential curve (see EM1 in Table 3), 95% of the variance to ED0 is focused on the first data point, and the imputed systematic error is around 1.8 mm Hg (i.e. 7.3 mm Hg (EDO) minus 5.5 mm Hg (EM1)). While the computational model estimates for the 95% confidence interval of the pressure at time zero just overlap with the 95% confidence interval of the measured experimental mean pressure at time zero (see Table 5), the cubic polynomial regressions and exponential regressions are not, which taken together support the hypothesis that there is a systematic error in the initial experimental data point.

Next we fit data set ED1 to a cubic polynomial regression (PM1), and then in a second step we fit the computational model to the PM1 regression curve. This prediction is labelled PCM1 in Tables 2, 4 and 6. We see that the predicted outflow parameters for the computational model are again pressure-dependent, but their values have now increased further (i.e. $C_{T}^{SL}$ is increased by a further 142% to 2.15 μl/min/mm Hg, *α* by a further 127% to 0.14 mm Hg^-1^ and the predicted outflow is increased by 118% to 9.0 μl/min; see Table 2).

**Table 6:** For ocular rigidity *C*_0_ equal to 45.5, the time variation of mean IOP predictions (based on the Eq 4), predicted by the computational model fitted to various data sets.

Time	ED0	CM0	PCM0	ECM0	ED1	CM1	PCM1	ECM1	ED1*
	Expt data	prediction	prediction	prediction	Expt data	prediction	prediction	prediction	Adjusted expt data
0	7.3	7.22	7.21	6.92	removed	6.35	5.92	5.72	6.35
5	9.3	9.57	9.60	9.89	9.3	9.37	9.28	9.32	9.3
10	12	11.70	11.72	12.03	12	11.78	11.82	11.85	12
15	13.4	13.41	13.40	13.52	13.4	13.51	13.55	13.53	13.4
20	14.6	14.66	14.63	14.56	14.6	14.69	14.71	14.64	14.6
25	15.3	15.51	15.46	15.27	15.3	15.48	15.48	15.39	15.3
30	16.1	16.06	16.02	15.78	16	16.00	15.98	15.89	16.1
35	16.6	16.42	16.38	16.13	16.6	16.35	16.32	16.24	16.6

The data sets are: EDO, the original data; ED1, is the reduced data set, that is the original data with the initial time point removed; ED1* is the ED1 data set with the first data point replaced by the CM1 model prediction. CMO is computational model fitted to data ED0. PCM0 is the computational model fitted to the cubic polynomial regression on ED0. CM1 is computational model fitted to ED1. PCM1 is computational model fitted to cubic polynomial regression on ED1. ECM1 is computational model fitted to exponential regression on ED1.

We can now explore the behavior of the model as constraints are put on the optimization process. For this investigation, we use data set ED1*, which is reduced data set ED1 with the initial data point replaced by the prediction by analysis CM1 (ED1* represents our ‘best estimate’ data set).

Using data set ED1* (see Table 7) to optimize the computational model, fixing *α* and ocular rigidity, and then allow $C_{T}^{SL}$ and the IOP at time zero to be the two optimization variables. From this, we can examine how the variance and predicted outflow varies with *α* (see Table 7). This is of interest because we found using both Dastridou et al. [26] and Karyotakis et al. [27] data sets that the mean value for *α* was around 0.07 between 20 mm Hg and 40 mm Hg, and so one might expect a similar value for *α* could be extrapolated to lower pressures.

Examining Table 7, we see that for an ocular rigidity of *c*_0_ equals 35.3 and *α* equals 0.07, then $C_{T}^{SL}$ is predicted to be 0.65 and the predicted outflow is 4.7 μl/min. For *c*_0_ equals 45.5 and *α* equals 0.07, then $C_{T}^{SL}$ is predicted to be 0.84 and the predicted outflow is 6.1 μl/min. We observe these two estimates are reasonably close to the previous estimates made Smith et al. [19] (e.g. outflow equals about 6.3 μl/min) and Smith et al. (2019) (outflow equals 6.0 μl/min).

**Table 7:** For ocular rigidity *C*_0_ equals 45.5 (or 35.3) and fixed *α*, optimized $C_{T}^{SL}$ solutions (based on Eq 4) and outflows predicted by the computational model fitted to data set ED1*.

*c*_0_ = 45.5							
*α*	0.0	0.04	0.07	0.075	0.08	0.10	0.15
S	0.35	0.27	0.22	0.21	0.21	0.19	0.23
$C_{T}^{SL}$	0.32	0.55	0.84	0.90	0.96	1.27	2.52
outflow	4.5	5.2	6.1	6.2	6.4	7.1	8.7
*c*_0_ = 35.3							
*α*	0.0	0.04	0.07	0.075	0.08	0.10	0.15
S	0.35	0.27	0.22	0.21	0.21	0.19	0.23
$C_{T}^{SL}$	0.24	0.43	0.65	0.70	0.75	0.99	1.96
outflow	3.4	4.1	4.7	4.8	5.0	5.5	7.3

**Fig 3:**
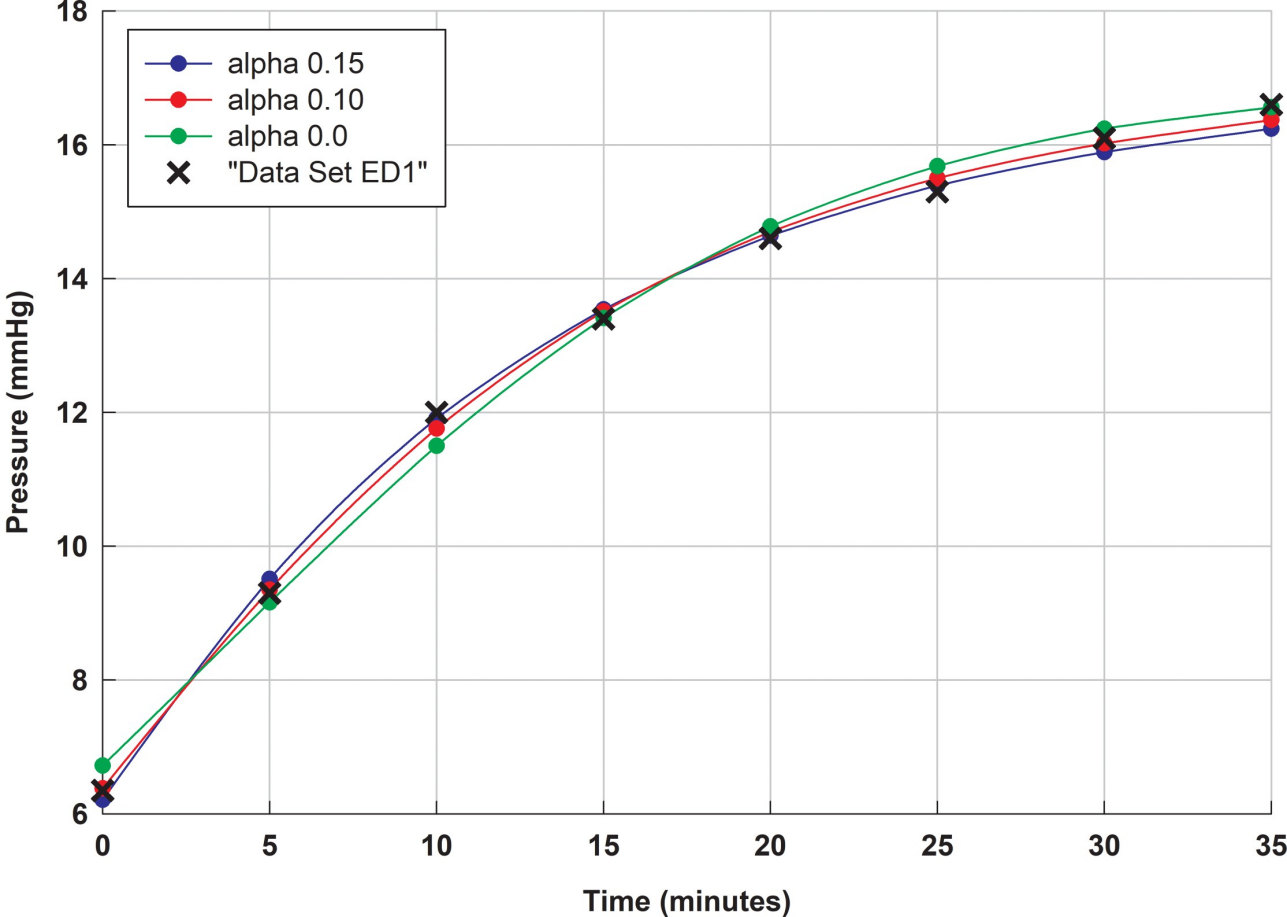
Comparison of computational model fits to data set ED1* (purple asterisks) (*C*_0_ equals 45.5). Computational model fits optimized to *α* equals 0.0 (green line), 0.10 (red line) and 0.15 (blue line). We note that to accurately estimate *α*, one needs to be able to discriminate between these three computational model curve fits, which have very different parameters (see Table *5*).

*S* denotes standard error of the regression. Note: pressure-time curves for *C*_0_ equals 45.5 and *α* equal 0.075 and 0.15 are shown in Fig 3.

## Discussion

When we developed the pressure dependent outflow model for the human eye, we explained that the model for the whole eye represented all the outflow pathways from the eye (i.e. the main outflow pathways being the trabecular pathway, the uveoscleral pathway, and the retinal pigment epithelium pathway) [19]. A ‘generalized model’ for the human eye that is the ‘sum of the individual outflow pathways’, is considerably more complex to parameterize, as each pathway has its own set of three parameters (i.e. 9 parameters rather than 3 parameters employed in [19]). The degree of model parameterization required for such a generalized model is well beyond the available data to test it. Consequently, we reduced the number of parameters in our pressure dependent outflow model for the human eye, reducing it to the smallest number of parameters possible (i.e. 3 parameters). In other words, we have effectively treated the individual outflow pathways as having similar pressure dependent modeling parameters, which are the same as the average pressure dependent outflow parameters for the whole eye.

We also pointed out that the uveoscleral pathway probably has significantly different parameters to the trabecular and retinal pigment epithelial pathways [19], because this pathway is said to be ‘pressure independent’ above about 10 mm Hg, but pressure dependent below this pressure [20] (e.g. we have previously estimated that perhaps *α* equals 0.25 for the uveoscleral pathway [19]). In other words depending on the magnitude of flow through uveoscleral pathway, if the IOP is ‘pushed down’ well below 10 mm Hg, there may be comparatively a very rapid decrease in pressure-dependent flow through the uveoscleral pathway, which may significantly influence computational model parameter estimates. Given the estimated size of *α* for the uveoscleral pathway, for IOP below about 10 mm Hg, one would expect this would bias the estimated pressure-dependent outflow parameters for the whole eye towards larger *α* values. These observations suggest that the homogenized computational model described in Smith and Gardiner [19], is probably best suited to analyzing data for the human eye at IOPs greater than about 10 mm Hg. This reasoning would suggest that the computational outflow model employed here is probably best tested from around 10 mm Hg and up to the normotensive pressure at 17 mm Hg (note that the first data point 7.3 mm Hg and the second data point is 9.3 mm Hg). By this reasoning, at least the first data point should be excluded in the fitting process, as it is likely to be at a pressure where the computational model is expected to be less accurate, at least for the pressure dependent outflow model in its current form. The second data point is probably sufficiently close to 10 mm for the model accuracy to be considered satisfactory (i.e. the error associated with this is probably of similar magnitude to measurement error).

In the results section, by quite separate reasoning, we chose to fit the computational model to a reduced data set (ED1), based on excluding the first data point. Interestingly, fitting the computational model to the reduced data set is consistent with the abovementioned limitations of the whole eye model (as further described in [19]), but in the Results section we did this for two main reasons: (i) the difficulty of obtaining measurements at time zero without incurring some delay in measurement, and (ii) following removal of the Honan Balloon after 30 minutes of application, it is reasonable to suppose the eye would temporarily be in a comparatively abnormal state.

So for these two main reasons, we chose to give most weight to the computational model predictions found by fitting the ED1 data set. Optimization of model parameters suggests that for *c*_0_ equals 45.5, $C_{T}^{SL}$ is about 1.5 μl/min/mm Hg, *α* is 0.11 and the outflow is 7.6 μl/min. For *c*_0_ equals 35.3, $C_{T}^{SL}$ is about 1.2 μl/min/mm Hg, *α* is 0.11 and the outflow is 5.9 μl/min. Importantly, these estimates are reasonably close to the estimates reported by Smith et al. [19]; viz, (i) for *c*_0_ equals 45.5, $C_{T}^{SL}$ is about 1.6 μl/min/mm Hg, *α* is 0.07 and the outflow is 10.9 μl/min (based on Dastiridou et al.’s data [26]) and (ii) for *c*_0_ equals 35.3, $C_{T}^{SL}$ is about 1.0 μl/min/mm Hg, *α* is 0.07 and the is outflow 6.3 μl/min (based on Karyotakis et al.’s data [27]).

During the analysis, we came to realize some features that provide valuable insights into fitting procedures. First and perhaps most importantly, we found when fitting the computational model that: (i) for a given intraocular pressure-time curve, the parameter $C_{T}^{SL}$ is almost entirely a function of the ocular rigidity, and (ii)*α* is almost entirely a function of the shape (or curvature) of the monotonic pressure-time curve between two pressures. For example, we note that as *α* increases, so the initial curvature of a monotonically increasing pressure-time curve also increases (see Fig 3). These observations were made when *c*_1_ equals zero, but they appear to be a good approximation if *c*_1_ takes a value of say 0.2 (i.e. a non-zero value for *c*_1_ in the ocular rigidity only confers a comparatively small function dependence on the other parameter). Practically this means that for conventional manometric testing of the eye, accurately defining the shape of the pressure-time curve is crucially important for identifying model parameter *α*, while accurately identifying the ocular rigidity is crucially important for defining model parameter $C_{T}^{SL}$. These observations serve to highlight the importance of accurately measuring both the ocular rigidity and the pressure-time curve if one is to accurately estimate the total outflow at the normotensive pressure. We note here that the total outflow also depends on a third model parameter,*p*_*T*_ (the no outflow pressure), which is taken as our best estimate throughout this paper to be 3 mm Hg, as found and discussed in Smith et al. [19]).

Next we observe that the ‘double fitting’ procedure resulted in a comparatively worse fit compared to directly fitting the computational model. For example, compare the standard error of 0.22 mm Hg for the computational model (CM0) fit to the raw data (ED0) (see Table 5) versus the standard error 0.29 mm Hg (i.e. $\sqrt{0.27^{2}+0.11^{2}}$, see Table 5) for the fitting of a cubic polynomial regression (PM0) followed by the computational model fit to the raw data (PCM0)). For the computational model directly fitted to the data, this results in a 95% confidence interval for the predicted mean to be about 0.48 mm Hg, versus a 95% confidence interval of about 0.64 mm Hg for the predicted mean for the double fitting procedure. We note a similar trend of additional total variance in later double-fitting procedures, so we conclude the double fitting procedure is probably sub-optimal compared to directly fitting the computational model to the experimental data.

We estimated the initial data point by fitting the computational model to the reduced data set (ED1), the intercept being the initial data point. Using this method, the predicted initial data point is about 1.0 mm Hg lower than the measured IOP. Consideration of variance of the prediction relative to the original data set (ED0) showed that the majority of the variance is focused on the first data point. This observation, together with similar conclusions being drawn for cubic polynomial and exponential regression curves, and being cognizant of the difficulty of obtaining a measurement at time zero, the biological plausibility of a temporarily abnormal and rapidly changing physiological state immediately following removal the Honan balloon (which had been in place for 30 minutes), all together build a case for the first data point having a systematic error.

There is no doubt the computational model parameters predicted are very sensitive to small changes in the data set, meaning that all findings made in this paper must be regarded as provisional. For example, compare model parameter estimates fitted to raw data set ED0 with the model parameters fitted to the adjusted data set ED1* (i.e. which have only about 1 mm Hg difference in the first data point; see Table 5). The parameter *α* changes from 0.11 (ED1* data) to about 0.03 (ED0 data set), and $C_{T}^{SL}$ from about 1.25 to 0.37, while the predicted outflow reduces from 7.6 μl/min to 4.5 μl/min. In other words, the sensitivity of *α* to uncertainty in the first data point is approximately (in units of mm Hg^-2^),

$$\frac{\Delta \alpha }{\Delta p_{t=0}}=\frac{\left(0.11-0.027\right)}{0.95}=0.087$$ (5)

Further evidence of sensitivity to parameter estimation is the effect of initial smoothing using a cubic regression curve, shown in Table 2 (i.e. *α* increases from 0.027 to about 0.035, $C_{T}^{SL}$ from 0.42 to 0.47 and outflow from 4.5 μl/min to 4.7 μl/min). It is clear that small changes in the data points lead to very different optimized parameter estimations, so we now consider the difficulty of accurately inferring outflow parameters from the pressure-time curve.

The reported standard deviation (*s*) of the individual sample measurements is shown Fig 1 (which ranges between 1.3 to 1.7 mm Hg). The standard error of the mean (SEM) is $\sqrt{s/n}$, so ± 2 × SEM is around 0.8 mm Hg, as shown in Fig 2). After fitting the computational model to the raw data (EDO), we find the standard error of the regression is 0.22, so the 95% confidence interval for the regression prediction of the mean is about 0.44 mm Hg. This implies that the uncertainty of each predicted mean data point after fitting the computational model’s curve is about one half the uncertainty of each mean value assessed individually.

After fitting the cubic polynomial regression to the raw data (EDO), we find the standard error of the regression is 0.27, so the 95% confidence interval for the regression prediction of the mean is about 0.54 mm Hg. We can then fit the regression predictions to the computational model (PCM0), and we find an additional standard error of the regression about 0.11, so the 95% confidence interval for the regression prediction of the mean is about 0.22 mm Hg (see Table 5). However the total standard error of the two regressions is about 0.29, so the 95% confidence interval for the regression prediction of the mean is about 0.58 mm Hg. In this example, the double fitting procedure has resulted in a worse fit compared to directly fitting the computational model (compare 0.44 mm Hg for the computational model fit to the raw data versus 0.58 mm Hg for the cubic regression followed by the computational model fit). Based on this evidence, we conclude the double fitting procedure is sub-optimal compared to directly fitting the computational model to the data.

The difficulty of accurately estimating computational model parameters from pressure-volume and pressure-time curves can be conveniently illustrated by fitting the computational model subject to constraints. Fig 3 shows plots of the best fit computational model curves to the data set ED1* (Table 6), assuming the outflow parameter alpha (*α*) is fixed to just three values 0.15, 0.10 and 0.0. For *α* equals 0.10, the ‘mean absolute error’ between the ED1* data points and the computational model predictions at each data time point is 0.13 mm Hg, and the standard error for the computational model is 0.19 mm Hg, meaning the 95% confidence interval for the prediction of a mean is about 0.38 mm Hg.

If we now consider *α* equals 0.0 (which in effect means the outflow hydraulic conductivity for the eye ($C_{T}^{SL}$) is constant, and so pressure independent), the mean absolute error between the ED1* data points and the computational model predictions at each data time point is only 0.22 mm Hg (about double 0.11 mm Hg found for *α* equals 0.1, but nevertheless still small). In other words, to fix the outflow parameters accurately, the standard error of the mean for a group of subjects needs to be much less than 0.22 mm Hg. What is apparent from Fig 3 is that to discriminate between these three solutions accurately requires that the standard error for the measurement be at least less than 0.05 mm Hg. Achieving such accurate estimates is not straightforward, and generally means invasive eye techniques are required.

Using manometric measurement (i.e. a 21 gauge needle with pressure transducer is placed into the anterior chamber of the eye,), Dastridou et al. [26] and Karyotakis et al. [27] employed a needle pressure sensor that had an accuracy of ± 0.13 mm Hg and a resolution of 0.05 mm Hg (converting resolution to a standard deviation equivalent, the ‘resolution uncertainty’ of the pressure sensor is $0.05/\sqrt{3}=0.03$ mm Hg). However the IOP is not constant *in vivo*. Indeed there exists a very obvious ocular pulse amplitude (OPA) [26], driven by vascular compliance and the pressure oscillation associated with systole and diastole of the heart, as well as a longer period pressure variation due to volume changes of the lungs (and heart rate) associated with breathing (the variation in heart rate with breathing is known as ‘sinus arrhythmia’) [28].

For the control group, OPA is reported by Dastiridou et al. to be 2.26 ± 0.63 mm Hg at 15 mm Hg, which increases by 0.066 ± 0.043 mm Hg for each mm rise in IOP, so the OPA at 40 mm Hg is 4.04 ± 1.03 mm Hg [26]. The pressure sensor employed by Dastridou et al. [26] and Karyotakis et al. [27] has a sampling rate of 200 Hz and a resolution of 0.05 mm Hg, and so the pressure sensor records the OPA variation easily. Consequently the raw data needs to be smoothed—we note that for intraocular pressure measured using a Goldmann applanation tonometer, the apparatus itself acts as a very low pass filter, smoothing the data. Karyotakis et al. explain that they smoothed the pressure-time recording by fitting an exponential curve to the raw pressure-time curve (the results of an exponentially decreasing curve fit to one subject’s pressure-time data curve is shown in figure 1 of Karyotakis et al. [27]).

Using this instrumentation and 19 subjects, Karyotakis et al. were able to measure the pressure-time curve and the pressure-volume curves for each individual, and find the interquartile range (i.e. middle 50% of measurements) for the local outflow facility (see Table 2 in [27]). Based on these interquartile range measurements, and assuming $C_{T}^{SL}$ equals one (which is close that reported by Karyotakis et al. [27]) we estimate the range for *α* is then about 0.06 to 0.09. Alternatively, if *α* is held constant at the mean value of 0.07, we estimate $C_{T}^{SL}$ ranges between about 0.7 and 1.3. Of course, some combination of *α*and $C_{T}^{SL}$ in these ranges may also be reasonably accurate solutions.

**Fig 4:**
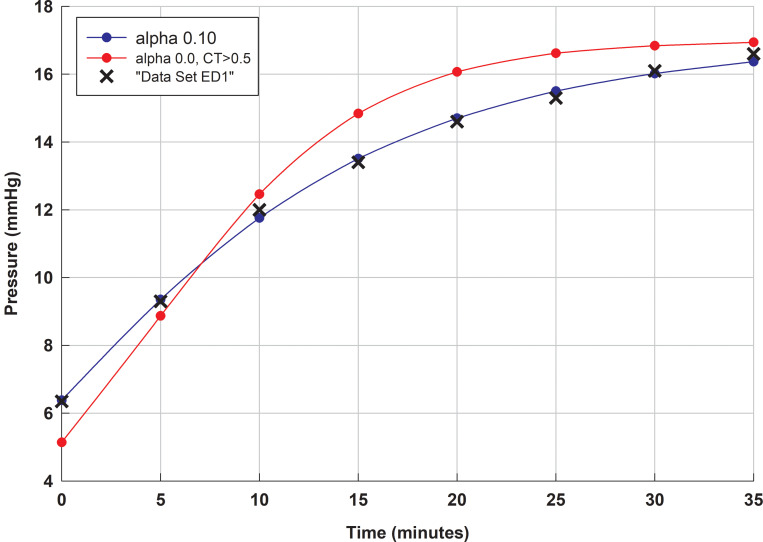
Comparison of computational model fits to data set ED1* (orange asterisks) (*C*_0_ equals 45.5). Computational model fits optimized to *α* equals 0.15 (blue) and *α* equals 0.0, subject to the constraint that $C_{T}^{SL}$ is greater than say 0.5 μl/min/mm Hg (red).

In this context, we further observe that if additional information is known about the ‘normal range’ for computational model parameter values, and this information can be formulated as a constraint on the computational model’s expected parameter range, this can greatly assist parameter estimation from a given pressure-time data set. For example, if the constraint is known that the surface hydraulic conductance ($C_{T}^{SL}$) for a normal human eye is greater than say, 0.5 μl/min/mm Hg, then upon fitting a regression curve with *α* equal to 0.0, it is clear that the optimal regression curve subject to this constraint is not a very good fit at all to Ernest et al.’s pressure-time curve (see Fig 4).

If for example, we now assume that for normal aged eyes that $C_{T}^{SL}$ ranges between about 0.7 and 1.3, we may seek as range for *α* that optimally fits Ernest et al.’s data (i.e. ED0 (or, in brackets, Ernest’s et al.’s modified data set ED1*)). If we do this, we find the expect range for *α* is 0.063 (0.058) to 0.11 (0.10). The expected range of outflow rates is between 5.4 μl/min (5.7 μl/min) and 7.4 μl/min (7.3 μl/min). We note both these ranges for *α* includes 0.07, which is approximately the mean value reported by Karyotakis et al., while the range for outflow rates include 6.0 to 6.3 μl/min, the expected values reported by Smith et al. [19] and Smith et al. (2019).

We observe that the accuracy of model parameter estimation is improved by continuous pressure measurement, more accurate IOP measurement (i.e. better pressure resolution), larger pressure changes, and longer periods of recording. Providing the eye tissues are not damaged by the pressure change, repeated measurements would also improve the accuracy of the curve fit.

Taking this analysis forward, we mention there are emerging technologies for injecting or withdrawing fluid from the eye at a constant rate, while rapidly establishing a steady state IOP [29, 30]. This means constant flow tests can now be done much more quickly than was possible previously, which may make it much more feasible to do studies on animal and human eyes. If several accurate constant flow tests were done in the human eye (perhaps two tests below and two tests above normotensive pressure), it would be possible to analyze the data (e.g. using Eq (1)), and so estimate the outflow parameter *a* from the ratios of the outflows at different pressures, and then together with data on the magnitude of the outflows, estimate the parameter $C_{T}^{SL}$.

Our outflow model may improve: (i) the interpretation of outflow measurements (which the present study expands), (ii) the interpretation of measured drug effects on fluid flow in the eye [16], and (iii) vitreal transport modeling, including the distribution of drugs within the vitreous humor following intravitreal injection [16]. Each of these has clinical implications, and we note that a discussion of the clinical implications can be found in the recent paper by Smith et al. [16].

## Conclusion

In this paper we have tested the pressure dependent outflow model formulated in Smith et al. [19] using a pressure-time experimental data at IOPs below the normotensive pressure in the human eye [21]. We chose to omit the first data point from the computational model fit, as it seems likely the experimental measurement at time zero is less accurate than measurements at subsequent times for a variety of reasons described above. Using this reduced experimental data set, the computational model parameters estimated by optimization of variance were *α* equals 0.11, $C_{T}^{SL}$ lies in the range 1.2 to 1.5 and outflow is in the range 5.9 μl/min to 7.6 μl/min. Though these estimates are reasonably close to the estimates made previously by Smith et al. [19], it is important to note that the estimates are very sensitive to small changes in the experimental data. This means very accurate pressure measurement are required to construct accurate pressure-volume and pressure-time curves, which are essential for accurate computational model parameter estimation by the method described here. We observe that the accuracy of parameter estimation is improved by continuous pressure measurement, larger pressure changes, and longer periods of recording. Providing the eye tissues are not damaged by the pressure change, repeated measurements are expected to also improve the accuracy of model parameter estimation.
